# What the Papers Say

**DOI:** 10.1093/jhps/hnu002

**Published:** 2014-06-12

*Journal of Hip Preservation Surgery* (*JHPS*) is not the only place that work in the field of hip preservation may be published. Although our aim is to offer the best of the best, we continue to be fascinated by work that finds its way into journals other than our own. There is much to learn from it, so *JHPS* has selected six recent and topical articles for those who seek a brief summary of what is taking place in our ever-fascinating world of hip preservation. What you see here are the mildly edited abstracts of the original papers, to give them what *JHPS* hopes is a more readable feel. If you are pushed for time, what follows should take you no more than 10 min to read. So here it goes … 

## IS INTRAOPERATIVE FLUOROSCOPY ANY USE FOR DELINEATING THE CAM DEFORMITY?

An interesting study from Michigan, Minnesota, New York (USA) and Zurich (Switzerland) has recognized the difficulties some surgeons may have in achieving what might be regarded as a full resection of a cam deformity during hip arthroscopy. The authors acknowledged that in the diagnosis and surgical treatment of cam-type femoroacetabular impingement (FAI), three-dimensional (3D) imaging is the gold standard for detecting femoral head–neck junction malformations preoperatively. Intraoperative fluoroscopy is used by many surgeons to evaluate and verify adequate correction of the deformity.

They had two purposes: (i) to compare radial reformatted computed tomography (CT) scans with six defined intraoperative fluoroscopic views before surgical correction to determine whether fluoroscopy could adequately depict cam deformity and (ii) to define the influence of femoral version on the clockface location of the maximum cam deformity on these views.

To do this they undertook a Level 2 cohort study and investigated a consecutive series of 50 hips (48 patients) that underwent arthroscopic treatment for symptomatic FAI by a single surgeon. Each patient underwent a CT scan and six consistent intraoperative fluoroscopy views: three views in hip extension and three views in hip flexion of 50°. The alpha angles of each of the fluoroscopic images were compared with the radial reformatted CT using a 3D software program. Femoral version was also defined on CT studies. Statistical analysis was performed using the Student *t*-test.

Of the 48 patients, 52% were male, with a mean age of 28 years (15–56). The maximum mean alpha angle on fluoroscopy was 65° (37°–93°) and was located on the anteroposterior (AP) 30° external rotation (ER) fluoroscopy view. In comparison, the mean CT-derived maximum alpha angle was 67° and was located at 1:15 o’clock (*P* = 0.57). The mean clockface positions of each of the fluoroscopy views (standardized to the right hip) were AP 30° internal rotation, 11:45 o’clock; AP 0° (neutral) rotation, 12:30 o’clock; AP 30° ER, 1:00 o’clock; flexion/0° (neutral) rotation, 1:45 o’clock; flexion/40° ER, 2:15 o’clock; and flexion/60° ER, 2:45 o’clock. Increased femoral anteversion (>20°) was associated with a significant change in the location of the maximum alpha angle (1:45 versus 1:15; *P* = 0.002).

The authors concluded that the described six fluoroscopic views are very helpful in localization and visualization of the typical cam deformity from 11:45 to 2:45 o’clock and can be used to reliably confirm a complete intraoperative resection of cam-type deformity in most patients. These views correlate with preoperative 3D imaging and may be of even greater importance in the absence of preoperative 3D imaging [1].

## T2 VALUES FOR EARLY DEGENERATION–WHAT IS NORMAL?

From Colorado (USA) has come an intriguing article on the subregional distribution of T2 values of articular cartilage in asymptomatic hips. The authors state that a standardized definition of normative T2 values across the articular surface of the hip must be defined to fully understand T2 values for detecting early degeneration. Therefore, in this article, they sought to lay a foundational methodology for reproducible quantitative evaluation of hip cartilage damage using T2 mapping to determine the normative T2 values in asymptomatic individuals. Nineteen prospectively enrolled asymptomatic volunteers (aged 18–35 years, males 10, females 9, alpha angle 49.3° ± 7.2°) and evaluated them with a sagittal T2 mapping sequence at 3.0 T magnetic resonance imaging. Acetabular and femoral cartilage was manually segmented directly on the second echo of the T2-mapping sequence by three raters, twice. Segmentations were divided into 12 subregions modified from the geographic zone method. Median T2 values within each subregion were compiled for further analysis and interrater and intrarater reliability were assessed. Their results showed that in the femur, the posterior–superior subregion was significantly higher (*P* ≤ 0.05) than those in the posterior–inferior and anterior–inferior subregions. In the acetabulum, the anterior–inferior subregion was significantly higher (*P* ≤ 0.001) than in the anterior–superior, middle, and posterior–inferior subregions. T2 values of the posterior–superior subregion were significantly higher (*P* ≤ 0.05) than the anterior–superior, middle, and posterior–inferior subregions. Interrater agreement was generally fair to good [2].

## SEVERE CHONDROPATHY MEANS A BAD RESULT

Meanwhile the effects of chondropathy on outcomes after surgery for FAI have been further clarified by work from Brisbane, Melbourne, Hobart (Australia) and Reading (UK). A study was undertaken to describe the prevalence of chondropathy in adults who had undergone hip arthroscopy for hip pain. The relationships between the severity of chondropathy and (i) participant characteristics and (ii) patient-reported outcomes (PROs) at initial assessment (mean 18 months post-surgery) and over a further 12 months (mean 30 months post-surgery) were evaluated. Finally, the relationships between chondropathy and coexisting FAI and labral pathology at the time of surgery were evaluated. The authors took 100 consecutive patients (age 36 ± 12 years) who underwent hip arthroscopy 18 months previously. Hip Osteoarthritis and Disability Outcome Score (HOOS) and International Hip Outcome Tool (iHOT-33) data were collected prospectively at 18 and 30 months post-surgery. Surgical data were collected retrospectively. Participants were grouped: outerbridge grade 0, no chondropathy; outerbridge grade I–II, mild chondropathy; outerbridge III–IV, severe chondropathy. The presence of FAI or labral pathology was noted. The prevalence of chondropathy (≥grade I) at hip arthroscopy was 72%. Participants with severe chondropathy were significantly worse for all HOOS subscales and the iHOT-33 at 18 months post-surgery [HOOS-symptoms (*P* = 0.017); HOOS-pain (*P* = 0.024); HOOS-activity (*P* = 0.009); HOOS-sport (*P* = 0.004); HOOS-quality-of-life (*P* =  0.006); iHOT-33 (*P* = 0.013)] than those with no chondropathy. At 12-month follow-up, HOOS-quality-of-life in those without chondropathy was the only PRO that improved. Relative risk of coexisting chondropathy with labral pathology or FAI was 40%. From this the authors concluded that chondropathy was prevalent, and associated with increasing age, coexisting labral pathology or FAI. Severe chondropathy was associated with worse pain and function at 18 months post-surgery. Little improvements were observed in participants over a further 12 months, regardless of chondropathy status [3].

## HIP ARTHROSCOPY IS HARDER THAN IT LOOKS

Hip arthroscopy is known to be a technically challenging procedure, so the length of the learning curve is clearly of interest. Authors from Pforzheim (Germany) have reported on this with a Level III study that aimed to determine whether the learning curve of arthroscopic treatment of FAI could be verified by analysing the complication rate of this procedure. Additionally, it was investigated whether supervision by an experienced surgeon leads to a steeper learning curve (lower number of complications) when starting to perform arthroscopic FAI treatment.

The complications occurring in 317 consecutive patients treated with the sole diagnosis of FAI were analysed. Two hundred fifty-six patients (collective A) were treated by surgeon A between June 2005 and January 2010. Sixty-one patients (collective B) were treated by surgeon B between August 2008 and December 2009. From January to June 2008, surgeon B performed many hip arthroscopies under the supervision of surgeon A. Complications were recorded in a central complication register. Statistical analysis of the complication rates was performed using Fischer's exact *t*-test. Subdividing collective A chronologically into thirds, a significant decline in complications (*P* = 0.0044) was found with growing experience of the surgeon. Comparing the first 61 patients of both surgeons, a significantly lower complication rate was discovered in the patients of surgeon B (*P* = 0.0375). In total there were 21 complications (6.6%; confidence interval 4.4–9.9%). The observed complication rate was 7.0% in collective A and 4.9% in collective B. The learning curve can be seen by the distribution of complications in collective A. Having spent 6 months performing under the supervision of surgeon A, surgeon B had a lower complication rate than surgeon A when comparing the first 61 patients each surgeon operated on. This implies that surgeon B benefited from the experience of surgeon A. According to this analysis, beginners in arthroscopic FAI treatment should be taught at a specialized center to reduce the number of complications [4].

## UNDERSTANDING THE LIGAMENTS OF THE HIP

As the debate surrounding stability of the hip joint gathers pace, work published from Seattle (USA) is particularly interesting. Quantitative descriptions of the hip joint capsular ligament insertional footprints have been reported. Using a three-dimensional digitizing system and computer modeling, the area and dimensions of the three main hip capsular ligaments and their insertional footprints were quantified in eight cadaver hips. The iliofemoral ligament (ILFL) attaches proximally to the anterolateral supra-acetabular region (mean area = 4.2 cm^2^). The mean areas of the ILFL lateral and medial arm insertional footprints are 4.8 and 3.1 cm^2^, respectively. The pubofemoral ligament (proximal footprint mean area = 1.4 cm^2^) blends with the medial ILFL anteriorly and the proximal ischiofemoral ligament (ISFL) distally without a distal bony insertion. The proximal and distal ISFL footprint mean areas are 6.4 and 1.2 cm^2^, respectively. The hip joint capsular ligaments have consistent anatomical and insertional patterns. Quantification of the ligaments and their attachment sites may aid in improving anatomical repairs and reconstructions of the hip joint capsule using open and/or arthroscopic techniques [5].

## SORRY, BUT YOU HAVE TO REPAIR THAT LABRUM

For those surgeons who hope to escape by not repairing the labrum at arthroscopy, an article from Houston (USA) is worth considering. The authors recognized that validated models of the hip that allow the quantification of labral function in functional joint positions have yet to be developed. Consequently they decided to evaluate two things by means of a descriptive laboratory study: (i) whether intra-articular pressures within the hip are regulated by fluid transport between the labrum and femoral head and (ii) whether the sealing capacity of the labrum varies with joint posture. To do this, the sealing ability of the hip labrum was measured during fluid infusion into the central compartments of eight cadaver specimens. Additionally, the pathway of fluid transfer from the central to the peripheral compartment was assessed via direct visualization in three specimens. The effect of joint posture on the sealing capacity of the labrum was determined by placing all eight specimens in 10 functional postures. The relationship between pressure resistance and three-dimensional motion of the femoral head within the acetabulum was quantified using motion analysis and computer modeling.

The results were fascinating. Resistance to fluid transport from the central compartment of the hip was directly controlled by the labrum during loading. Maximum pressure resistance was affected by joint posture (*P* = 0.001). Specifically, positions that increased ER of the joint (pivoting) provided an improved seal, while positions that increased flexion combined with internal rotation (stooping) augmented the ease of fluid transport from the central to the peripheral compartment. Maximum pressure resistance was associated with the distance between the labrum and femoral head during pivoting. This study demonstrated that the transfer of fluid from the central compartment of the hip occurs at the junction of the labrum and femoral head. Joint position was shown to strongly affect the sealing function of the labrum and was attributable to the distance between the labrum and femoral head in certain positions. It seems that altering the relationship between the labrum and femoral head may disrupt the sealing ability of the labrum, potentially leaving the joint at risk for pathological changes with time [6].

## References

[1] Ross JR Bedi A Stone RM Intraoperative fluoroscopic imaging to treat cam deformities. Correlation with 3-dimensional computed tomography. Am J Sports Med 2014. 10.1177/0363546514529515 24737661

[2] Ho CP Surowiec RK Ferro FP Subregional anatomical distribution of T2 values of articular cartilage in asymptomatic hips. Cartilage 2014. 10.1177/1947603514529587 PMC4297181 26069695

[3] Kemp JL Makdissi M Schache AG Hip chondropathy at arthroscopy: prevalence and relationship to labral pathology, femoroacetabular impingement and patient-reported outcomes. Br J Sports Med. 10.1136/bjsports-2013-093312 24659505

[4] Dietrich F Ries C Eiermann C Complications in hip arthroscopy: necessity of supervision during the learning curve. Knee Surg Sports Traumatol Arthrosc 2014; 22: 953-8. 24519620 10.1007/s00167-014-2893-9

[5] Telleria JJ Lindsey DP Giori NJ A quantitative assessment of the insertional footprints of the hip joint capsular ligaments and their spanning fibers for reconstruction. Clin Anat 2014; 27: 489–97. 24293171 10.1002/ca.22272

[6] Dwyer MK Jones HL Hogan MG The acetabular labrum regulates fluid circulation of the hip joint during functional activities. Am J Sports Med 2014; 42: 812–9. 24557859 10.1177/0363546514522395

